# Conditional Knockout of Tumor Overexpressed Gene in Mouse Neurons Affects RNA Granule Assembly, Granule Translation, LTP and Short Term Habituation

**DOI:** 10.1371/journal.pone.0069989

**Published:** 2013-08-06

**Authors:** Elisa Barbarese, Marius F. Ifrim, Lawrence Hsieh, Caiying Guo, Vedakumar Tatavarty, Michael J. Maggipinto, George Korza, Jessica W. Tutolo, Anthony Giampetruzzi, Hien Le, Xin-Ming Ma, Eric Levine, Brian Bishop, Duck O. Kim, Shigeyuki Kuwada, John H. Carson

**Affiliations:** 1 Department of Neuroscience, University of Connecticut Health Center, Farmington, Connecticut, United States of America; 2 Department of Molecular, Microbial and Structural Biology, University of Connecticut Health Center, Farmington, Connecticut, United States of America; 3 Department of Genetics and Developmental Biology, University of Connecticut Health Center, Farmington, Connecticut, United States of America; Western University of Health Sciences, United States of America

## Abstract

In neurons, specific RNAs are assembled into granules, which are translated in dendrites, however the functional consequences of granule assembly are not known. Tumor overexpressed gene (TOG) is a granule-associated protein containing multiple binding sites for heterogeneous nuclear ribonucleoprotein (hnRNP) A2, another granule component that recognizes *cis*-acting sequences called hnRNP A2 response elements (A2REs) present in several granule RNAs. Translation in granules is sporadic, which is believed to reflect monosomal translation, with occasional bursts, which are believed to reflect polysomal translation. In this study, TOG expression was conditionally knocked out (TOG cKO) in mouse hippocampal neurons using cre/lox technology. In TOG cKO cultured neurons granule assembly and bursty translation of activity-regulated cytoskeletal associated (ARC) mRNA, an A2RE RNA, are disrupted. In TOG cKO brain slices synaptic sensitivity and long term potentiation (LTP) are reduced. TOG cKO mice exhibit hyperactivity, perseveration and impaired short term habituation. These results suggest that in hippocampal neurons TOG is required for granule assembly, granule translation and synaptic plasticity, and affects behavior.

## Introduction

TOG, the product of the CKAP5 gene, is a long filamentous protein comprised of reiterated TOG domains, each consisting of multiple HEAT repeats [1], [2]. TOG was originally identified as a microtubule binding protein [3] that regulates microtubule growth [4] and is important for spindle formation in mitotic cells [5], [6]. Subsequently TOG was shown to bind to hnRNP A2 [7] a cognate *trans*-acting RNA binding protein that recognizes *cis*-acting sequences called hnRNP A2 response elements (A2REs) in dendritically localized RNAs encoding proteins. These include: α calcium/calmodulin-dependent protein kinase (αCaMKII), neurogranin (NG), activity-regulated cytoskeleton associated (ARC) protein, and protein kinase M zeta (PKMξ), all of which are required for regulation of synaptic function in neurons [8]. A2RE RNA molecules are incorporated into trafficking/translation intermediates called RNA granules that are translocated to dendrites [9], [10] where they are locally translated. Granule translation is generally sporadic (<2 translation events per 10 sec), which is believed to reflect monosomal translation, with occasional periods of bursty translation (>3 events per 10 sec), which are believed to reflect polysomal translation [11]. Assembly of A2RE RNA into granules is thought to be mediated by sequence specific binding of A2RE RNAs to hnRNP A2 molecules and multivalent binding of hnRNP A2 molecules to TOG protein. To investigate the functional consequences of mRNA assembly into granules in neurons, TOG expression was conditionally knocked out in hippocampal neurons using cre/lox technology and the effects on granule assembly, local translation, synaptic plasticity and behavior were investigated. ARC RNA was used as a reporter RNA for granule assembly and translation in control and TOG cKO neurons because it contains an A2RE sequence, is incorporated into granules and exhibits bursty translation in hippocampal neurons [8], [11]–[13]. ARC protein mediates endocytosis of α-amino-3-hydroxy-5-methyl-4-isoxazolepropionic acid receptor (AMPAR) [14], [15] so expression and trafficking of AMPAR subunit GluA1 were used to report on ARC function in control and TOG cKO hippocampal neurons. Field excitatory post synaptic potentials (fEPSPs) and long-term potentiation (LTP) were measured in the hippocampal CA1 region of brain slices from control and TOG cKO mice because granule RNAs (such as ARC RNA) encode proteins that regulate synaptic function in hippocampal neurons. Hippocampus-associated behavioral phenotypes including marble burying [16]–[18], locomotion activity in the open field test and ultrasonic vocalizations (USVs) in response to olfactory stimuli, were analyzed in control and TOG cKO mice. Our results indicate that TOG expression in neurons affects granule assembly, bursty translation, synaptic function and behavior.

## Materials and Methods

### Ethics statement

Use of animals as described in this study followed the guidelines of the University of Connecticut Health Center (UCHC) Animal Care Committee (Animal Welfare Assurance number A-3124-01) and those of the National Institutes of Health and was approved specifically by our Institutional Animal Care and Use Committee: the UCHC Animal Care Committee.

### Animals

The following strains of mice were obtained from The Jackson Laboratory (Bar Harbor, MA): B6.Cg-Tg(Camk2a-cre)T29-1Stl/J (Stock # 005359) [19], (B6.129X1-*Gt(ROSA)26Sor^tm1(EYFP)Cos^*/J) (Stock # 006148), 129S1/Sv-*Hprt^tm1(cre)Mnn^*/J (Stock # 004302), 129S4/SvJaeSor-*Gt(ROSA)26Sor^tm1(FLP1)Dym^*/J (Stock # 003946) and C57BL/6 (Stock # 000664). Floxed TOG mice and TOG*^+/null^* mice were produced at the UCHC Gene Targeting and Transgenic Facility (GTTF).

### Generation of a floxed TOG (aka CKAP5) mouse

**Figure 1:**
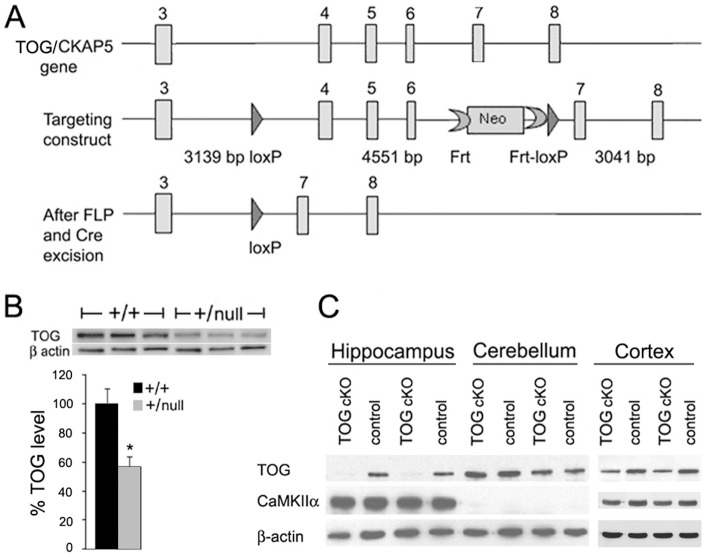
TOG knockout in hippocampal neurons. A. Diagram of the portion of the *TOG/CKAP5* gene that is targeted for excision, and the targeting construct. The bottom panel shows the out-of-frame deletion after *Cre* excision. B. Western blot of brain homogenates from +/+ (control) and +/null mice stained with rabbit anti-TOG and mouse anti-β-actin; error bars indicate standard deviations; n = 6 animals for each genotype (t-test *p<0.05). C. Western blot of homogenates from hippocampus, cerebellum and cortex of 2 month old control (wild type) and TOG cKO mice stained with rabbit anti-TOG, mouse anti-αCaMKII and mouse anti-β-actin; n = 3 animals for each genotype.

The targeting vector (described in Fig. 1A) was electroporated into D1 ES cells derived from F1 hybrid blastocyst of 129S6× C57BL/6J at the GTTF. G418-resistant ES colonies were isolated and screened by nested polymerase chain reaction (PCR) using primers outside the construct paired with primers inside the neo cassette. Primer sequences were as follows: 5′ arm forward primers: TOG Scr F1 (5′- tgcacctccccattgtatga -3′) and TOG Scr F2 (5′- cccacatgtaagacaggtac-3′); reverse primers: LoxP scrR1 (5′–gagggacctaataacttcgt-3′) and loxP scrR2 (5′-ggaattgggctgcaggaatt-3′); 3′ arm forward primers: frt scr F1 (5′-ttctgaggcggaaagaacca-3′) and frt scr F2, (5′-cgaagttattaggtggatcc-3′); reverse primers: TOG Scr R1 (5′- agtacttcaggccaagtgtct -3′) and TOG Scr R2 (5′- gtcttgggaagagctagact -3′). Six clones that were PCR-positive for both arms were expanded and the genotype confirmed after ES cell expansion. Chimeric mice were generated by aggregating ES cells with 8-cell embryos of CD-1 mice. The neo cassette was removed by mating the chimeras with 129S4/SvJaeSor-*Gt(ROSA)26Sor^tm1(FLP1)Dym^*/J (Stock #: 003946) homozygous females. F1 pups were genotyped by PCR with primers flanking the loxP site or frt-loxP site. The primer set lox gtF (5′- cctagcacttgggatactga -3′) and lox gtR (5′- ggctagcctcaatttcatgc -3′) generated PCR products of 258 bp for the wild type allele and 352 bp for the floxed allele. The primer set frt gtF (5′- tttgtgtcctggacaccgta -3′) and frt gtR (5′- ggcctatttgctacctagag -3′) generated PCR products of 222 bp for the wild type allele and 317 bp for the floxed allele. After *CRE* excision the knockout allele was detected by PCR with lox gtF and frt gtR primers. The length of PCR product for knockout allele is 287 bp. The wild type allele is too long to be amplified under these conditions. Template DNA was obtained by digesting tail sample (0.2–0.5 mm) or ear piece in 50 µl proteinase K buffer (50 mM Tris pH8.8, 1 mM EDTA pH 8.0, 0.5% Tween-20 and proteinase K 0.6 mg/ml) at 55°C overnight followed by heat inactivated at 100°C for 10 minutes. An aliquot (0.8 μl) of the digest was used as template in a PCR reaction (20 μl) carried out for 30 cycles (94°C 30 s, 55°C 30 s and 72°C 30 s) followed by one cycle of 72°C for 5 min. Floxed TOG mice were backcrossed with C57BL/6 mice (>9 generations) to generate C57Bl/6 congenic floxed TOG mice. TOG*^+/null^* mice were generated through germline knockout using the 129S1/Sv-*Hprt^tm1(cre)Mnn^*/J driver line [20]. TOG cKO mice were generated by mating floxed TOG mice with αCaMKII-*CRE* driver line [B6.Cg-Tg(Camk2a-cre)T29-1Stl/J (Stock # 005359)] whose *Cre* recombinase expression is reportedly restricted to hippocampal CA1 neurons [19].

### Western blot

Gel electrophoresis and western blotting were performed as described [7] using the following antibodies: chicken anti-TOG (1∶1,000) [7], rabbit anti-CKAP-5 (TOG) (1∶5,000) (GeneTex, # GTX30693), rabbit anti-CKAP5 (1∶5,000) (QED Bioscience, # 34032), rabbit anti-TOG (1∶1,000) (a generous gift from Dr. Christian Larroque, INSERM, Montpellier, France), rabbit anti-GluA1 (1 µg/ml) (abcam, # ab31232), anti-CamKII (1∶20,000) (monoclonal clone 6G9, Millipore, # MAB8699), anti-β-actin (1∶10,000) (monoclonal clone AC-15, Sigma, # A5441) and appropriate horseradish conjugated secondary antibodies (1∶10,000) (Jackson ImmunoResearch).

### Cell Surface Protein Analysis

Cell surface proteins on hippocampal neurons were labeled and isolated using Pierce Cell Surface Protein Isolation Kit # 89881. Cells were labeled with membrane-impermeable thiol-cleavable amine-reactive biotinylation reagent. Following lysis with mild detergent, biotin labeled proteins were isolated with NeutrAvidin Agarose beads. Avidin bound proteins were eluted by incubating with SDS-PAGE sample buffer containing 50 mM DTT and analyzed by western blot with rabbit anti-GluA1 (1 μg/ml) (abcam).

### Cell culture

Hippocampal neurons from E19-P0 mice were cultured as described in [21] with modifications as described in [8].

### Histo- and immuno-chemistry

Tissue sections and cultured cells were fixed with 4% paraformaldehyde, permeabilized with detergent, incubated successively with blocking reagent, primary and secondary antibodies. Antibodies include: affinity-purified chicken anti-TOG (1∶50) (Aves Labs, Tigard, OR, under contract), rabbit anti-CKAP5 (1∶250) (GeneTex), mouse anti-NeuN (1∶250) (Millipore), mouse anti-MAP2 (1∶100) (Zymed) and secondary conjugated antibodies from Molecular Probes (Carlsbad, CA). Nuclei were stained with TO-PRO-3 (1∶500) (Invitrogen) and f-actin was stained with Texas Red conjugated phalloidin (1∶20) (Invitrogen).

### Hippocampal neuron morphology and spine density in culture and in brain sections

Neurons in culture were stained with Texas Red conjugated phalloidin and imaged by fluorescence confocal microscopy using a 63 X, 1.4 NA objective. Spine densities for control and TOG cKO neurons were analyzed without prior knowledge of sample genotype. Neurons in hippocampal sections were stained with TO-PRO-3 and/or anti-NeuN and imaged by fluorescence confocal microscopy. The width of the CA1 stratum pyramidale was measured at five positions in each brain section and averaged. For determining spine density and morphology, animals were perfused with 3% paraformaldehyde. CA1 hippocampal neurons were labeled by ballistic delivery of DiI according to the protocol in [22] with modifications as described in [23]. Z-stack images through the apical dendrites of CA1 pyramidal neurons were collected by fluorescence confocal microscopy using a 63 X, 1.4 NA objective. Maximum intensity Z-stack images were analyzed using MetaMorph software as described in [23]. The length of the dendrites in each image was measured and the number of spines was counted and their morphologies classified according to [24]. Types A and B spines have no head and a shaft longer than 1.3 μm. Type B spines have a thicker shaft than type A. Types C and D spines have a mushroom head and a shaft longer than 0.7 μm. Type D spines have a thicker shaft than type C. Types F and G spines are stubby with a shaft shorter than 0.7 μm. Type G spines have a thicker shaft than type F spines. Quantification of control and TOG cKO samples was performed without prior knowledge of the genotype of the animals.

### Recombinant TOG protein and TOG domain peptides

Full length human TOG cDNA (in pBSII KS+ vector) was obtained from Dr. T. Nagase (Kazusa DNA Research Institute, Japan). TOG-GFP was prepared by PCR using a forward primer (5′-ctccaccgcgggattacaaggaaaacctggaa-3′) to introduce a SacII site and a reverse primer (5′-gcgggatcctttgcgactgctctttattc-3′) to introduce a BamH1 site flanking the TOG sequence. The amplified product was digested with SacII and BamH1 and subcloned into pEGFP-C1 (Clontech). Recombinant TOG-GFP was expressed in pEAK Rapid cells (Edge Biosystems, Gaithersburg, MD) and purified using the μMACS Epitope Tag protocol with anti-GFP antibody (Miltenyi Biotec). Individual TOG domains (D1-D7), identified by secondary structure prediction, were amplified by PCR using the following primers:

D1 5′-atgggagatgacagtgagtgg-3′ (sense)

5′-ttatccaccagcagactgttgttg-3′ (antisense)

D2 5′-aaactcgaggatgctgaaggaggtggtgat-3′ (sense)

5′-ttacttagcagccaggtgcctttttT-3′ (antisense)

D3 5′-ccgctcgaggctggtgggccaccaaaaaag-3′ (sense)

5′-aaggaggatgaagacaaatcc-3′ (antisense)

D4 5′-cggctcgagaccagaggaatttccaagcat-3′ (sense)

5′-ttataagctggttttgcttggcat-3′ (antisense)

D5 5′-aaggaggatgaagacaaatcc-3′ (sense)

5′-ttagttggcattggagcttatgtt-3′ (antisense)

D6 5′-caccatgttacgcaagggaccagct-3′ (sense)

5′-ttaagggtccttatgggaaattca-3′ (antisense)

D7 5′-caccaagaccctgctacacacctta-3′ (sense)

5′-tcatttgcgactgctctttattctctc-3′ (antisense)

TOG domains D6 and D7 were cloned in pET100/D-TOPO expression vector (Invitrogen). TOG domains 1–5 were cloned into pCRT7/NT-TOPO vector (Invitrogen). Both expression vectors contain an N-terminal hexahistidine tag for purification of the recombinant fusion protein on a metal affinity column. Sequence and orientation for each construct were confirmed by complete DNA sequencing.

### Single molecule imaging of granule assembly and translation

RNA encoding Venus ORF fused upstream of ARC ORF and 3′UTR was transcriptionally labeled with Cy5-conjugated UTP (Amersham biosciences) followed by capping and polyadenylation using mScript Kit (Epicentre). Solubilized RNA was filtered through a 0.22 micron filter (Millipore) prior to microinjection. Concentration and intactness of labeled RNA was measured by fluorescence correlation spectroscopy (FCS) and agarose gel electrophoresis. Labeled RNA was microinjected into control or TOG cKO hippocampal neurons in culture. Granule assembly was analyzed by imaging the intracellular distribution of injected RNA by fluorescent microscopy. The number of Venus-ARC RNA molecules per granule was determined by comparing fluorescent intensities of individual granules to intensities of single RNA molecules immobilized on a glass slide imaged under identical conditions. Translational output per granule was analyzed by single molecule imaging of newly synthesized Venus-ARC protein molecules in the vicinity of each granule as described previously [11]. Each newly synthesized Venus-ARC molecule produces a flash of fluorescence, which is rapidly photobleached. Each flash corresponds to a single translation event. Centroid coordinates for each flash indicate spatial locations for each translation event. Centroid coordinates for newly synthesized protein molecules were superimposed on an image of fluorescent RNA granules to provide a translation event map. Times of first appearance for newly synthesized protein molecules associated with individual granules were recorded to provide translation event schedules for each granule. Sporadic translation is defined as a translation rate of <2 events/10 sec. Bursty translation is defined as a translation rate of >3 events/10 sec.

### Electrophysiology

Mice (2 months old) were anesthetized by 4% isoflurane inhalation and euthanized by decapitation. Parasagittal slices containing the hippocampus [25] were prepared in cold artificial cerebral spinal fluid (ACSF) containing (in mM) 124 NaCl, 3 KCl, 1.25 NaH_2_PO_4_, 1 MgSO_4_, 26 NaHCO_3_, 10 dextrose, 2 CaCl_2_, 0.4 ascorbate, 4 Na-lactate, 2 Na-pyruvate, and equilibrated with 95% O_2_ and 5% CO_2_ (290±5 mOsm ⋅ kg-1, pH 7.2). Brain slices (400 μm thick) were cut with a Dosaka EM DTK-1000 (Kyoto, Japan) and transferred to an incubation chamber containing oxygenated ACSF at room temperature (23–25°C) until recordings were performed (within 3 hours of slicing). All recordings were conducted in a submerged bath chamber continuously perfused (1.5 to 2 ml/min at room temperature) with ACSF containing GABAA antagonist (GABAzine, 5 μM) equilibrated with 95% O_2_ and 5% CO_2_. Field potential recordings were obtained from the stratum radiatum of hippocampal CA1 identified under infrared differential interference contrast video microscopy on an Olympus BX50W microscope equipped with 4× objective. Recording pipettes (2–4 MΩ), filled with the same ACSF solution used in the bath, were pulled from borosilicate glass capillaries using a Flaming/Brown P-97 (Sutter Instruments, Novato, CA) micropipette puller. Electrical recordings were made with an Axon 200B amplifier (Molecular Devices, Sunnyvale, CA), filtered at 2.9 kHz and digitized at >6 kHz. To evoke field potentials at 0.05 Hz, a bipolar tungsten stimulation electrode was placed in the stratum radiatum of hippocampal CA3 region. Stimuli were set to evoke 0.2 to 0.4 mV field responses during baseline period. LTP was induced by theta burst stimulation (TBS) consisting of 10 trains of 5 pulses (100 Hz) at theta frequency (5 Hz) [26].

### Behavior tests

Male control and TOG cKO mice were housed 2–3 to a cage on a 12 h light/dark cycle. Behavior tests were carried out during the light cycle. All tests, except for ultrasonic vocalization (USV) recording, were performed in the Scoville Neurobehavioral Suite (UCHC). USV recording was performed in a sound attenuated chamber. General health parameters (body weight, appearance of fur, body posture and gait) were monitored weekly. Neurological reflexes to tactile, visual and acoustic stimuli were tested approximately 1 week before administering all other tests as described in [27]. Animals were habituated to the test room for 1 h before administration of the tests. Balance, coordination and endurance were measured with a rotarod apparatus. Stride and gait were measured by coating the feet with food coloring to track each footprint as the animal walked towards an enclosed dark box along a track lined with white paper. Distance and alignment between consecutive footprints were analyzed [28]. Anxiety was measured by recording time spent in the open and enclosed quadrants of an elevated zero maze apparatus (San Diego Instruments) [29]. The marble burying test was performed using a clear plastic cage (19”×10.5”×6”) with 4.5 cm of bedding containing twenty black glass marbles (15 mm in diameter) evenly spaced in a 4×5 grid pattern covering 2/3 of the cage. At the beginning of the test the mouse was placed in the portion of the cage (1/3) without marbles and allowed to roam freely for 30 minutes, after which the number of marbles buried, how they were buried and their arrangement were recorded [16], [17]. The test was performed on 3 months old control floxed/floxed mice and TOG cKO mice. Contextual memory was evaluated in a passive avoidance test [30]. The test was performed using the Gemini Avoidance System (San Diego Instruments) that has a light and a dark chamber. The test consists of a training trial and a retention trial as described in [30], [31]. For the training trial, the mouse is placed in the light chamber for a 15 s habituation period. After that time, the chamber is illuminated and the gate is raised for 300 s to give the mouse access to the dark chamber. Latency to enter the dark chamber is recorded. After the mouse has crossed to the dark chamber the gate is closed and the mouse immediately received a 0.35 mA foot shock for 2 s. The mouse remains in the dark chamber for 30 s after the shock. The retention trial is performed 24 h after training. The procedure is the same as for the training trial. Three months old control (wild type) and 2.5 months TOG cKO mice were tested. Short term habituation to spatial stimuli was measured using the open field test (PAS Open Field system, San Diego Instruments) [30], [32]. Total numbers of infrared photobeam breaks per 5 minutes intervals were recorded over a period of 60 minutes. Two and a half months old control (wild type) and TOG cKO were tested. Habituation to female olfactory/pheromonal stimuli was measured by recording USVs emitted by a sexually naïve male (3 months old wild type control and TOG cKO mice) placed in the same cage with a female mouse in estrus but separated by a wire-grid barrier [33]. USVs were recorded in a sound attenuated chamber using a Brüel & Kjær 1/8” microphone and preamplifier (type 4138) coupled to a Brüel and Kjær amplifier and power supply (type 5935). Vocalizations were digitized at 200 kHz sampling rate using a digital signal processor (TDT RX6). Spectrograms were analyzed using Adobe Audition software. Individual USVs were classified as: monotonic upward, monotonic downward, short (<5 msec), chevron, inverted chevron, flat, complex, composite and step [34], [35]. Frequency ranges for individual USVs were classified as <72 kHz, –72 kHz -, and >72 kHz.

## Results

### TOG cKO in hippocampal neurons

Floxed TOG transgenic mice were produced using recombineering techniques [36]. A 10,373 bp genomic DNA fragment containing exons 3–8 of the *CKAP5* gene (encoding TOG protein) was retrieved from BAC clone RP23-118G11. A loxP sequence was inserted in intron 3 and a frt-PGKNeo-frt-loxP cassette was inserted in intron 6. Thus, a fragment of 4,551 bp genomic DNA encompassing exons 4 through 6 was floxed. The out of frame deletion resulting from the action of *CRE* recombinase is depicted in Fig. 1A.

To assess the effect of *CRE*-mediated deletion in the TOG (*CKAP*) gene, TOG*^+/null^* mice were generated through germline knockout using the 129S1/Sv-*Hprt^tm1(cre)Mnn^*/J driver line [20]. TOG expression in brain homogenates of control and +/null mice was measured by western blotting with antibodies to either the C terminus (Fig. 1B) or N-terminus (not shown) of human TOG protein. The intensity of the band corresponding to full length TOG protein (∼218 kDa), detected by either antibody, was decreased (by approximately 45%) in +/null brain homogenates compared to control (+/+) brain homogenates, indicating that *CRE*-mediated deletion of one copy of the TOG gene reduces overall TOG expression and suggesting that expression from the intact TOG locus does not compensate for the deleted locus.

Knocking out TOG in the whole animal may be embryonic lethal because TOG protein is ubiquitously expressed in all cells and is required for spindle formation during mitosis [6]. To determine if TOG null/null mice are viable, heterozygous TOG*^floxed^*/null mice were mated and seven litters (33 pups) were genotyped. The average litter size for TOG*^floxed^*/null x TOG*^floxed^*/null litters was 5 pups compared to 7 pups for control litters. Genotyping identified 11 TOG*^floxed^*/TOG*^floxed^*, 22 TOG*^floxed^*/null and 0 null/null mice, suggesting that TOG null/null mice are embryonic lethal, possibly because of the requirement for TOG in mitosis.

To knockout TOG conditionally in post-mitotic neurons, TOG*^floxed/floxed^* mice were crossed with transgenic mice that express *CRE* recombinase from the promoter for αCaMKII, which is expressed in CA1 hippocampal neurons beginning at approximately 21 days post-natal in the B6.Cg-Tg(Camk2a-cre)T29-1Stl/J mouse line [19]. In this specific line of αCaMKII-*CRE* mice, *CRE* expression is described as being restricted to the hippocampal CA1 region; however, its action has been reported to spread to other regions of the brain in older animals [37], [38]. In 2 months old TOG*^floxed/floxed^*; +/αCaMKII-*CRE* mice (TOG cKO), expression of TOG protein was very low in the hippocampus (where *CRE* is expressed), was not affected in the cerebellum (where *CRE* is not expressed) but was reduced by 48% in the cortex (where αCaMKII is present) (Fig. 1C). TOG cKO mice older than 4 months exhibited late onset morphological changes outside the CA1 region of the hippocampus, as well as behavioral deficits that may reflect TOG KO in other brain regions or secondary effects of impaired CA1 neurons on connected pathways. To avoid these confounding variables, subsequent experiments were performed with mice ≤3 months of age (after αCaMKII-*CRE* is expressed in CA1 neurons but before the appearance of morphological changes beyond the hippocampus). Mice with the TOG*^floxed/floxed^*; +/αCaMKII-*CRE* genotype are referred to as TOG cKO, whereas TOG*^floxed/floxed^* mice are referred to as control. The latter were indistinguishable from αCaMKII-*CRE*/αCaMKII-*CRE* or wild type mice in all of the tests used in this study (data not shown).

### Morphology of CA1 hippocampal neurons in TOG cKO mouse brain

**Figure 2:**
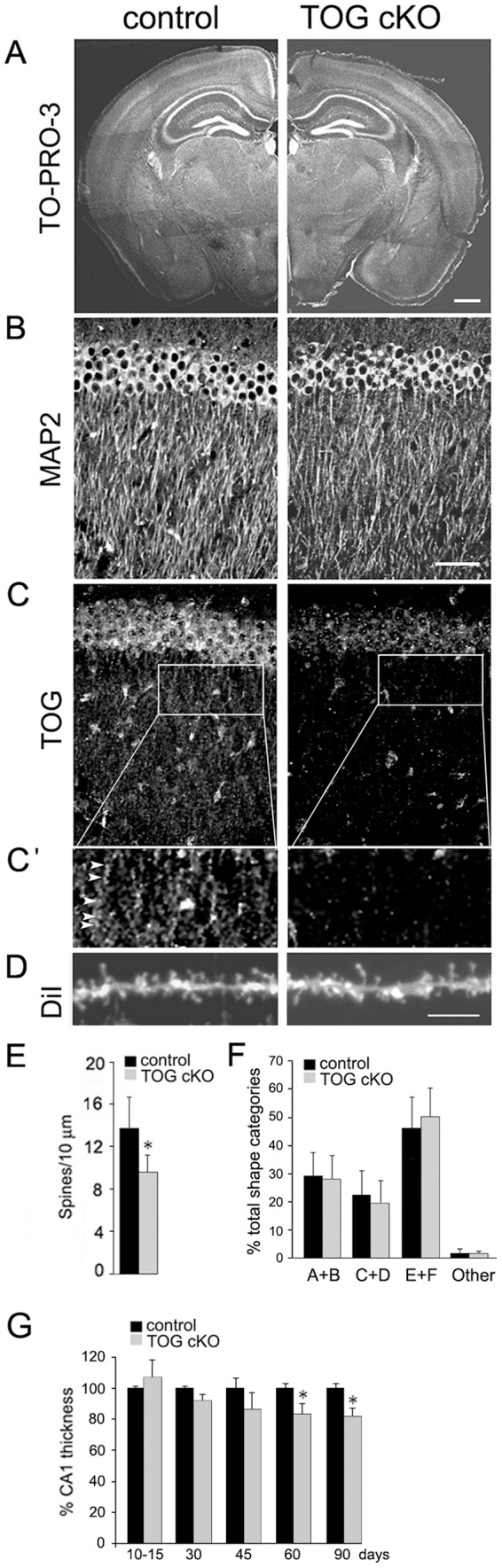
Neuronal and dendritic spine morphology in control and TOG cKO hippocampus. A. Coronal brain sections from 60 day control and TOG cKO mice stained with TO-PRO-3. The image montage for control and TOG cKO sections was assembled from multiple overlapping microscopic fields of the same tissue section. Scale bar  = 200 μm. B. Sections of hippocampal CA1 region from 60 day control and TOG cKO mice stained with anti-MAP 2. Scale bar  = 50 μm. C. Same sections as in B stained with anti-TOG. C'. Inset of the apical dendritic layer shows granules (arrowheads) in control but not in TOG cKO section. D. DiI labeled hippocampal CA1 dendrites in control and TOG cKO mice. Scale bar  = 2 μm. E. Quantification of the density of hippocampal CA1 apical spines in control and TOG cKO mice; n = 2 for each genotype. Twenty and 18 images were analyzed for control and TOG cKO, respectively. Error bars indicate standard deviations (t-test *p<0.01). F. Spine morphologies for control (909) and TOG cKO (696) spines classified according to [24]. Types A and B are filopodia-like, types C and D are mushroom-like, types F and G are stubby. Error bars indicate standard deviations (t-test p>0.05). G. Quantification of thickness of hippocampal CA1 stratum pyramidale in control and TOG cKO mice. Brain cross-sections stained with TO-PRO-3 and anti-NeuN were imaged by fluorescence confocal microscopy; n = 2 to 3 mice for each genotype at each time point. Error bars indicate standard deviations (t-test, *p<0.05).

The gross morphology of the CA1 region (visualized by staining with the nuclear dye TO-PRO-3) (Fig. 2A), and the organization of CA1 apical dendrites (visualized by immunostaining with antibodies to microtubule-associated protein 2 (MAP2) (Fig. 2B), were comparable in control and TOG cKO brain. In TOG cKO brain, TOG expression was reduced in the CA1 stratum pyramidale and apical dendritic layer (Fig. 2C). In control neurons TOG was localized in discrete granules (Fig. 2C' arrows) but in TOG cKO neurons the number of TOG-positive granules (Fig. 2C') was reduced. Spine density in apical dendrites of CA1 pyramidal neurons, determined by DiI labeling (Fig. 2D), was ∼70% of control in TOG cKO brain (Fig. 2E) (which is within the range reported previously for wild type mice of the same age [23]. Proportions of spines of different shapes, classified according to [24], were comparable in control and TOG cKO brain (Fig. 2F). In TOG cKO mice older than 45 days, there was a decrease (<20%) in the thickness of the CA1 stratum pyramidale (Fig. 2G) possibly due to cell loss. Loss in cell number and reduction in spine density may reflect neuronal pruning due to long term effects of TOG KO in the CA1 region of the hippocampus. No change in overall morphology was observed in other regions of the hippocampus up to 3 months. These results indicate that TOG KO in hippocampal neurons has little effect on the thickness of the CA1 stratum pyramidale and no significant effect on the morphology of apical dendrites and spines up to 2 months of age.

### TOG cKO hippocampal neurons in culture

**Figure 3:**
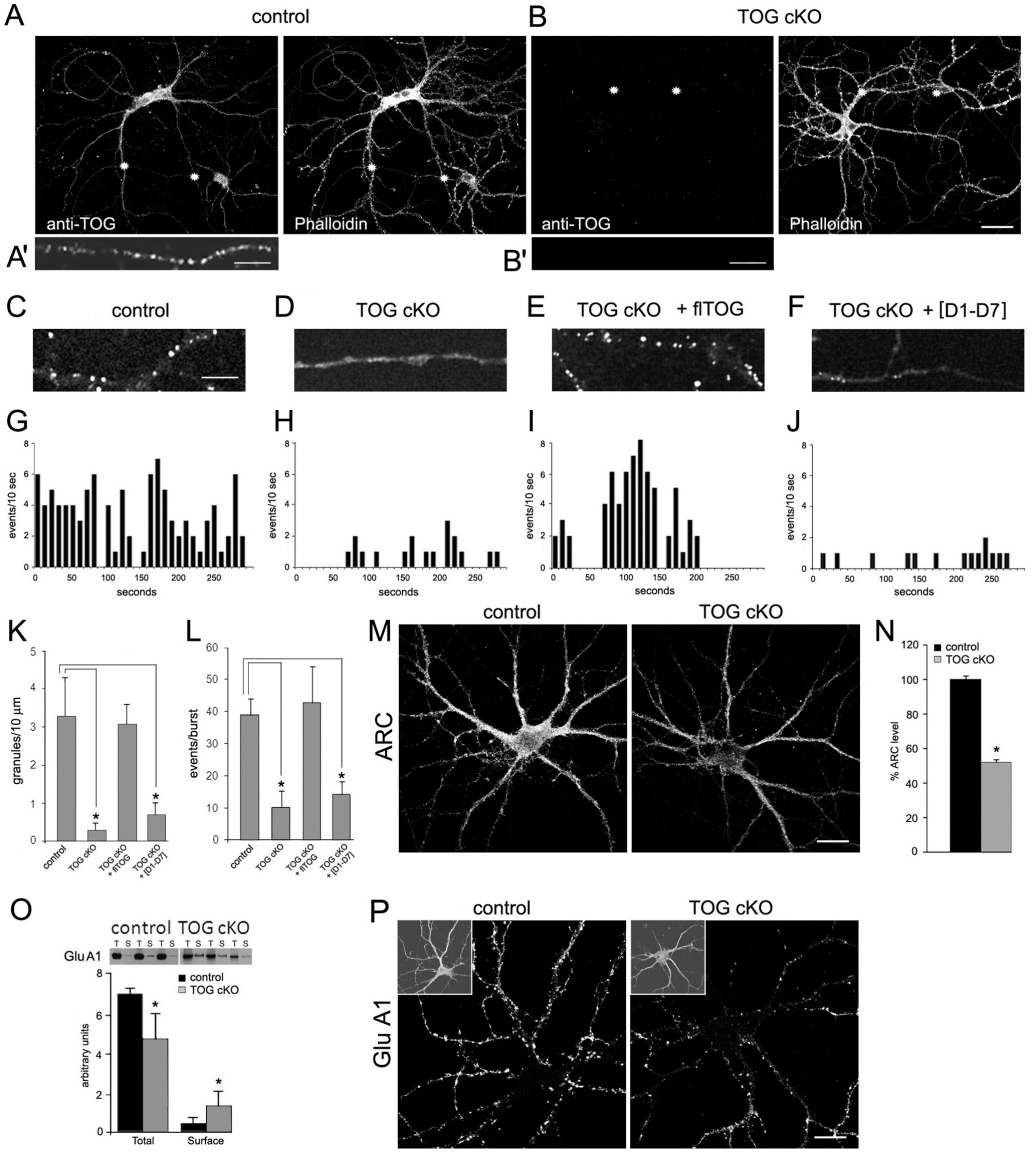
TOG expression, granule assembly, granule translation and ARC expression in control and TOG cKO hippocampal neurons in culture. A. Fluorescence microscopic images of a cultured control neuron co-stained with anti-TOG and Alexa 488 conjugated secondary antibody and Texas-red conjugated phalloidin to label f-actin. A'. High magnification of the dendritic segment identified by 2 asterisks in A and stained with anti-TOG. Scale bar  = 10 μm. B. Fluorescence microscopic images of 2 cultured TOG cKO neurons co-stained with anti-TOG and Alexa 488 conjugated secondary antibody and Texas-red conjugated phalloidin. B'. High magnification of the dendritic segment identified by 2 asterisks in B and stained with anti-TOG. Scale bar  = 10 μm. C – F. Subcellular distribution of microinjected Venus-ARC RNA (labeled with Cy5-UTP) in control (C) and TOG cKO (D) hippocampal neurons and in TOG cKO neurons co-injected with full-length recombinant TOG protein (E) or with an equal molar mixture of individual TOG domains [D1–D7] (F). Injected cells were visualized by wide field fluorescence microscopy. Scale bar for C – F = 10 μm. G – J. Number of translation events per 10 sec is plotted versus time. Representative translation profiles under conditions described in C – F. K. Numbers of Venus-ARC RNA containing granules were counted in 10 μm dendritic segments of neurons treated as in C – F. Values represent average and standard deviations for numbers of granules per 10 μm dendritic segments (t-test, *p<0.05). L. Numbers of translation events per burst were counted for individual granules in control, TOG cKO, TOG cKO plus full length TOG protein and TOG cKO plus TOG domains in cells injected with Venus-ARC RNA (labeled with Cy5-UTP). A burst is defined as a sustained period of elevated translation activity (>3 events/10 sec) preceded and followed by periods of lower translation activity (<2 events/10 sec). Values represent average and standard deviations for numbers of events per burst in different granules (t-test, *p<0.05). M – N. ARC protein levels were measured in control and TOG cKO neurons (n = 8) after immunostaining with anti-ARC and a fluorescent secondary antibody. Error bars indicate standard deviations (t-test; *p<0.05). O. Quantification of total (T) and surface (S) GluA1 in control and TOG cKO hippocampal neurons in culture using biotinylation and western blotting. Error bars indicate standard deviations (t-test; *p<0.05). P. Control and TOG cKO hippocampal neurons were stained with anti-Glu A1 after 18 days in culture. The insets in P are low magnification of the same cells stained for actin. Scale bar in M and P = 20 μm.

To identify cells expressing αCaMKII-*CRE* recombinase in culture, hippocampal neurons were cultured from TOG cKO mice carrying an expression cassette for enhanced Yellow Fluorescent Protein (EYFP) with an upstream *loxP*-flanked STOP sequence [39]. At 14 days in culture, EYFP was detected in 96% of hippocampal neurons from TOG cKO/EYFPloxPSTOP mice, indicating that, at this age in culture, *CRE* recombinase is expressed in most TOG cKO neurons. Neurons were immunostained with antibody to TOG protein to visualize TOG expression. TOG was detected in the perikaryon and dendrites of control neurons (Fig. 3A), but was not detected in TOG cKO neurons (Fig. 3B). In dendrites of control neurons, TOG was present in granules as seen in Fig. 3A'. TOG-positive granules were not detected in TOG cKO dendrites (Fig. 3B'). To visualize overall cell morphology and spine density, neurons were labeled with phalloidin, which binds to f-actin in spines, (Fig. 3A, B). The pattern of phalloidin staining was comparable in control and TOG cKO neurons at 16 days in culture, indicating that lack of TOG expression does not affect overall cell morphology or spine density (8 spines/10 μm, t-test, p>0.05).

### Granule assembly and translation of ARC RNA in TOG cKO hippocampal neurons in culture

To determine if TOG is required for granule assembly and translation, Cy5-labeled RNA encoding Venus-ARC was microinjected into the perikaryon of control and TOG cKO neurons in culture. Fluorescent (Cy5) Venus-ARC RNA is a reporter for granule assembly and newly synthesized fluorescent Venus-ARC protein is a reporter for translation events in individual granules. In control neurons Venus-ARC RNA was incorporated into granules localized to dendrites (Fig. 3C). Individual granules exhibited sporadic translation (<2 events/10 sec) interspersed with periods of bursty translation (>3 events/10 sec) (Fig. 3G). In TOG cKO neurons, the distribution of Venus-ARC RNA appeared more disperse, with fewer and smaller RNA granules localized to dendrites (Fig. 3D). Translation of Venus-ARC protein in TOG cKO neurons was sporadic (<2 events/10 sec) with reduced translation event frequency and few, if any, bursts (Fig. 3H). Both the density of Venus-ARC RNA granules (Fig. 3K) and the frequency of translation events (Fig. 3L) were significantly reduced in TOG cKO dendrites, indicating that TOG is required for granule assembly and bursty translation of ARC RNA.

To determine if TOG protein is sufficient for granule assembly and bursty translation, exogenous full-length recombinant TOG protein was co-injected with Venus-ARC RNA into TOG cKO neurons. Both granule assembly (Fig. 3E) and bursty translation (Fig. 3I) were rescued by full-length TOG protein. To determine if intact TOG protein is required for granule assembly and bursty translation, or if individual TOG domains are sufficient, an equimolar mixture of individual TOG domains was co-injected into TOG cKO neurons. Assembly into granules (Fig. 3F) and bursty translation (Fig. 3J) were not rescued by the mixture of individual TOG domains (Fig. 3K and L, respectively). This implies that granule assembly and bursty translation require that individual TOG domains are linked together in a multivalent scaffold in intact TOG protein. An alternative possibility is that granule assembly and bursty translation require a contiguous sequence in intact TOG protein that is disrupted when TOG domains are expressed individually. This possibility could be tested by injecting overlapping fragments of TOG, but this was not done in this study.

To determine if reduced bursty translation of ARC RNA in TOG cKO neurons affects steady state levels of ARC protein, control and TOG cKO neurons were immunostained for ARC protein (Fig. 3M). The overall intensity of ARC staining was reduced in TOG cKO neurons relative to control, indicating that reduced bursty translation of ARC RNA was associated with reduced steady state levels of ARC in TOG cKO neurons (Fig. 3N).

Since ARC protein mediates endocytosis of AMPAR [14], [15], [40] reduced expression of ARC protein in TOG cKO neurons may affect AMPAR trafficking. To test this possibility, surface proteins were biotinylated and total and surface GluA1 were quantified by western blotting (Fig. 3O) in control and TOG cKO neurons. Total GluA1 expression was reduced by ∼30% in TOG cKO neurons, suggesting that TOG regulates overall GluA1 expression. Despite the reduction in total GluA1 levels, surface GluA1 was increased ∼3 fold in TOG cKO neurons, indicating that endocytosis of GluA1 is reduced in TOG cKO neurons, which may be due to reduced expression of ARC protein. Total (surface and internalized) AMPARs were visualized by immunofluorescence in Fig. 3P. The overall distribution of AMPARs between cell body and dendrites was similar in control and TOG cKO neurons indicating that TOG KO does not affect overall dendritic localization of AMPAR.

### fEPSPs and LTP in TOG cKO hippocampal brain slices

**Figure 4:**
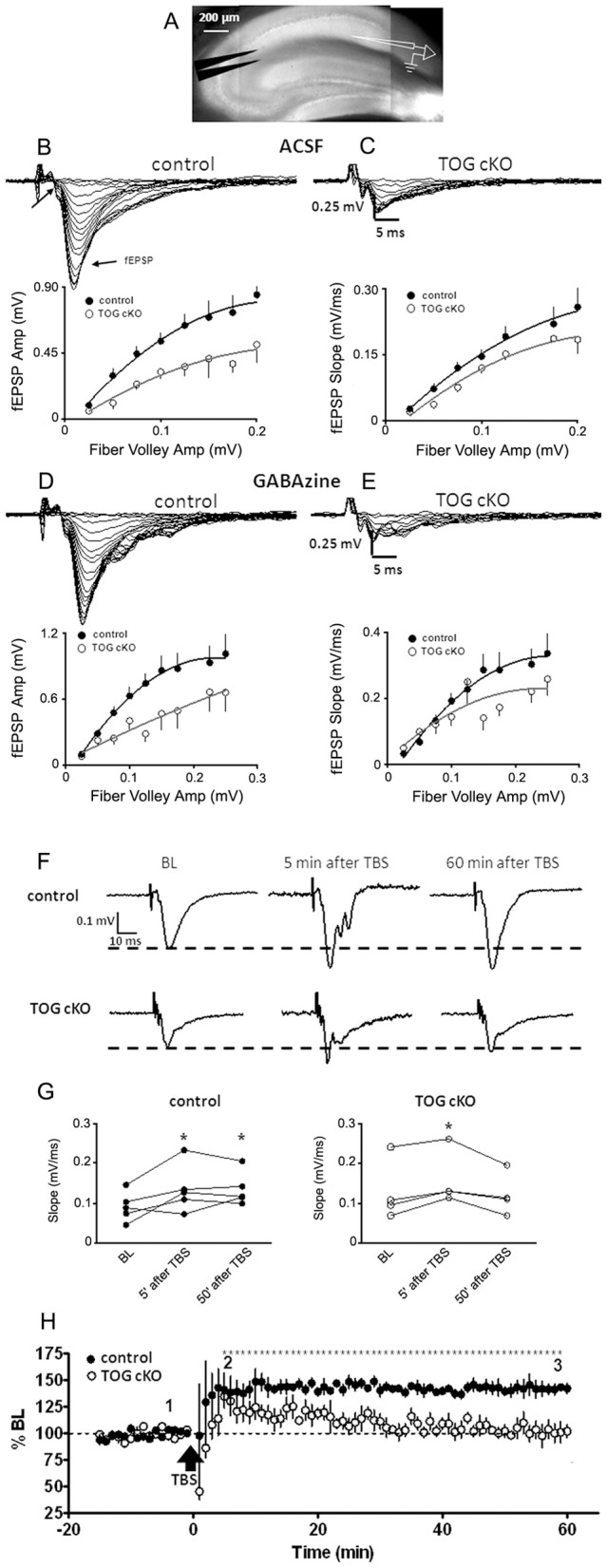
fEPSPs and LTP in CA1 of control and TOG cKO hippocampal brain slices. A. Infrared differential interference contrast micrograph of a hippocampal slice showing bipolar tungsten electrode (left) and glass recording electrode (right) placement. B – C. Electrical recording of field responses from control (B) and TOG cKO (C) slices at various stimuli intensities. Presynaptic fiber volley and field excitatory postsynaptic potential (fEPSP) are marked by arrows. Input output curves generated from control (n = 7 slices from 5 animals) and TOG cKO (n = 7 slices from 4 animals) plotted using fEPSP amplitudes (left) and rising slopes (right). Error bars represent standard deviation of the mean. D – E. Field responses and input output curves in the presence of GABAA antagonist (GABAzine, 5 μM) for the same specimens as in B and C. Error bars represent standard deviation of the mean. F. Electrical recording of field responses in control and TOG cKO hippocampal slices, in presence of GABAzine (5 μM), during baseline (BL) as well as 5 and 60 minutes after theta burst stimulation (TBS, 10 bursts in 2 sec). G. Rising slopes of fEPSPs during BL, and 5 and 60 minutes after TBS, from control (n = 5 animals) and TOG cKO (n = 4 animals). * denotes significant difference from BL (repeated measures ANOVA followed by Newman-Keuls multiple comparison test, p<0.05). H. Group time-course before and after TBS, normalized to the rising slopes of its corresponding BL. Error bars represent standard deviation of the mean.* denotes significant difference from BL period in control (repeated measures ANOVA followed by Bonferroni's multiple comparison test, p<0.05).

Reduced translation of Venus-ARC RNA (and possibly other granule RNAs) and reduced expression and endocytosis of GluA1 may affect synaptic function in TOG cKO mice. To measure basal synaptic function, field excitatory postsynaptic potentials (fEPSPs) were recorded from the CA1 region of the stratum radiatum in acute hippocampal slices from control and TOG cKO mice (Fig. 4A). Field responses were evoked by electrically stimulating Schaffer collateral axons that directly innervate CA1 neurons [41]. Input-output curves for TOG cKO hippocampal slices had reduced amplitudes (Fig. 4B) and slopes compared to control (Fig. 4C) indicating reduced basal excitatory transmission. GABAergic inhibition, which can potentially attenuate fEPSPs was blocked by GABA antagonist GABAzine (5 μM). Schaffer collateral stimulation in control and TOG cKO evoked larger field responses in the presence of GABAzine, as measured by the slopes of fEPSPs (Fig. 4D, E). However, field responses in the presence of GABAzine were still smaller in TOG cKO than control, indicating that this phenotype is not due to greater GABAergic tone in TOG cKO neurons. These results indicate that TOG cKO animals have reduced excitatory synaptic response in the stratum radiatum compared to control. This is consistent with both reduced dendritic spine density in TOG cKO neurons [42], as observed in Fig. 2D and E as well as reduced granule assembly and translation of granule RNAs in TOG cKO neurons, as observed in Fig. 3.

To test the ability of TOG cKO neurons to undergo long-term potentiation (LTP) of synaptic efficacy in response to normally potentiating electrical stimuli, the effect of theta burst stimulation (TBS) of the Schaffer collaterals on evoked field synaptic responses in acute hippocampal slices was compared (Fig. 4F) in control and TOG cKO slices. Evoked fEPSPs, recorded in the presence of GABAzine (5 μM), were potentiated 5 minutes after TBS in both control and TOG cKO slices. Potentiation was sustained up to 60 minutes in control slices but reverted to baseline levels by 30 minutes in TOG cKO slices. Due to contaminating population spikes, evoked fEPSP slopes were analyzed instead of amplitudes (Fig. 4G) [26]. Overall, both control and TOG cKO slices showed significant short-term potentiation in evoked field potentials at 5 minutes after TBS (control: 159±32% of BL, n = 5 animals, p<0.05; TOG cKO: 132±12% of BL, n = 4 animals, p<0.05). LTP was maintained for up to 60 minutes in control (159±24% of BL, p<0.05) but not in TOG cKO slices (100±8% of BL, p>0.05). Group time-course (Fig. 4H) indicated that TOG cKO were unable to maintain LTP of evoked potentials, which gradually returned to pre-TBS levels by 30 minutes, suggesting that TOG expression is required for maintenance of synaptic potentiation in hippocampal neurons. The LTP deficit in TOG cKO neurons is consistent with a deficit in ARC translation, since ARC expression is required for LTP. However other granule RNAs, which also encode proteins required for LTP, may also be affected in TOG cKO neurons. Thus, the LTP deficit in TOG cKO neurons could reflect disrupted translation of multiple different granule RNAs.

### Behavior in TOG cKO mice

TOG cKO mice were indistinguishable from control mice in tests for balance, coordination and endurance measured with a rotarod apparatus, stride and gait measured by footprint tracking, and anxiety measured in the elevated zero maze.

**Figure 5:**
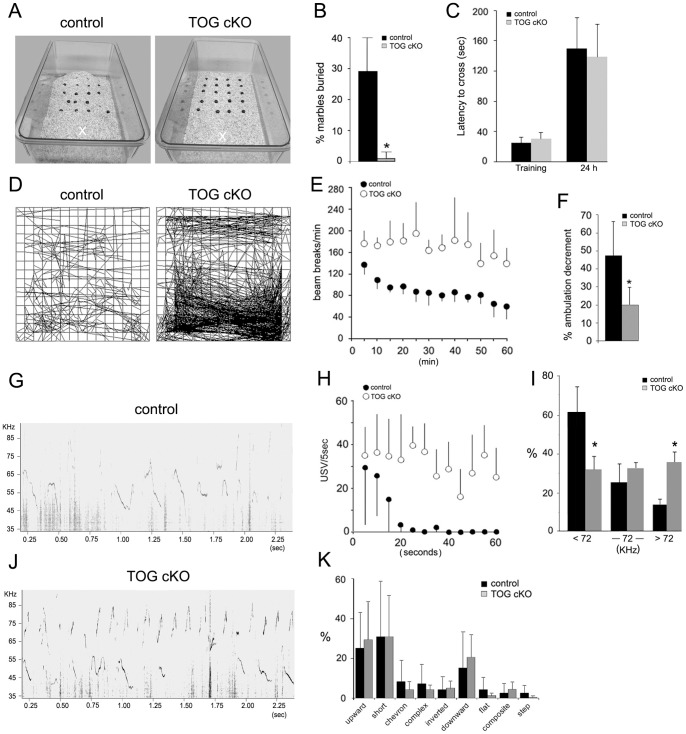
Behavioral phenotype of control and TOG cKO mice. A – B. Marble burying test. A. Marble distribution 30 minutes after a control (left) or a TOG cKO (right) mouse was placed in the cage at position indicated by X. B. Average numbers of marbles buried for both control and TOG cKO mice (n = 10/genotype). Error bars indicate standard deviations (t-test; *p≤0.01). C. Passive avoidance test. Average latency to cross from the light chamber into the dark chamber for the training trial (Training) and retention trial (24 h) conducted 24 h after the training trial (n = 10 mice/genotype). Error bars indicate standard deviations (t-test; p>0.05). D – F. Open field test. D. Representative ambulation patterns of control and TOG cKO mice. E. Average numbers of beam breaks per minute recorded at 5 minutes intervals are shown for 10 control and 7 TOG cKO males. Standard deviations for control and TOG cKO mice are indicated in opposite directions for clarity. F. Ambulation decrement: differences in ambulation scores in (E) between the first 15 minutes and the last 15 minutes of the observation period calculated as percentage of the first 15 minutes of observation; (t-test; *p≤0.05). G – K. USV recording for control and TOG cKO animals. Representative USVs patterns for control (G) and TOG cKO (J) mice. H. Average numbers of USVs recorded per 5 sec at 5 sec intervals are shown for control (n = 5) and TOG cKO (n = 5) males (ANOVA, F = 11.7; df = 1,110; p = 0.009). Standard deviations for control and TOG cKO mice are indicated in opposite directions for clarity. I. USV frequencies for control and TOG cKO mice. Percent of USVs below 72 KHz, spanning the 72 KHz arbitrary boundary, and above 72 KHz during 1 min of recording. K. USVs categories for control and TOG cKO mice.

Previous studies have shown that hippocampal damage is associated with reduced marble burying [18]. The marble burying test was used to measure a species-typical behavior (digging) that is in part associated with the hippocampus [17]. Representative results for control and TOG cKO mice are shown in Fig. 5A. Control mice buried approximately 30% of the marbles in 30 minutes, while TOG cKO mice buried few if any marbles (t-test, p<0.01) (Fig. 5B). These data indicate that the absence of TOG expression results in a hippocampus-related behavioral deficit.

The passive avoidance test was used to measure contextual memory. There was no difference in latency to cross to the dark chamber between control and TOG cKO mice during the training trial or 24 h later (Fig. 5C). This could be due to the fact that passive avoidance is not a pure hippocampus-dependent task, unlike other contextual learning paradigms; it has a significant fear component related to the amygdala [43].

The open field test [27] was used to measure locomotor activity and short term habituation to the novel spatial stimulus of the apparatus by recording decline in activity over time [32], [44], [45]. Representative ambulation patterns of control and TOG cKO mice are shown in Fig. 5D. When activity was recorded over a one hour period, TOG cKO mice exhibited approximately twice as much activity as control mice (175 vs 89 beam breaks/min, t-test p<0.001) (Fig. 5E). However, when activity was recorded over 5 minute intervals control and TOG CKO mice had comparable activity at time 0, but activity declined over time with control mice (indicating habituation) but not with TOG cKO (Fig. 5E–F). The decrease of activity over time was significantly different between control and TOG cKO (ANOVA, F = 2.4; df  = 11, 132; p<0.01). This indicates that absence of TOG expression in hippocampal neurons causes impaired short term habituation to spatial stimuli.

Functional magnetic resonance imaging (fMRI) studies have implicated the hippocampus in habituation to olfactory stimuli [46]. USV recording from a sexually naïve male mouse placed near a female mouse in estrus was used to measure short term habituation to sexual olfactory or pheromonal stimuli. Fig. 5G and J show representative results of USV recordings from a control and a TOG cKO mouse, respectively. Fig. 5H displays the rate of vocalizations over the first 60 seconds for control and TOG cKO mice. TOG cKO mice vocalized significantly more than control mice. However, when USVs were recorded over 5 sec intervals both control and TOG cKO mice had comparable USVs at time 0, but the rate of vocalizations declined rapidly for control mice and more slowly for TOG cKO mice, although the differences between the two groups did not reach statistical significance. Variations in the TOG cKO cohort data indicate that a larger sample size would be necessary to confirm a decrease in habituation.

The shapes of the FM sweeps for individual USVs recorded on the spectrograms of control and TOK cKO males were classified as described previously [34], [35]. Proportions of different classes of vocalizations are plotted in Fig. 5K. The overall repertoire of USV classes was not statistically different in control and TOG cKO mice. Frequency ranges for individual USVs were classified as low (<72 kHz), intermediate (continuous from below 72 kHz to above 72 kHz (−72 kHz -) or high (>72 kHz) (Fig. 5I) for control and TOG cKO mice. Control mice had higher proportions of low frequency (<72 kHz) USVs compared to TOG cKO mice, which had higher proportions of high frequency (>72 kHz) FM sweeps compared to control mice. This suggests that control mice have a low band-pass filter mechanism to suppress high frequency USVs and that this mechanism is somehow impaired in TOG cKO mice. Overall, the vocalization analysis indicates that absence of TOG in hippocampal neurons affects response to sexual or pheromonal olfactory stimuli. Alterations in USV classes have been reported in several mutant mouse models for autism in humans [35], [47]–[49].

## Discussion

TOG cKO in neurons has pleiotropic phenotypes affecting granule assembly, granule translation, synaptic function and behavior. We propose that the primary TOG-dependent phenotype is disruption of granule assembly and that other phenotypes reflect downstream granule-dependent processes. Previous work has shown that granule assembly requires molecular interactions between A2RE sequences and hnRNP A2 molecules [50]. Granule assembly may be TOG-dependent because TOG functions as a multivalent scaffold for hnRNP A2 molecules that bind to granule RNAs. It is not known if other hnRNP proteins that have been implicated in dendritic spine density and synaptodentritic mRNA metabolism [51], [52] also bind to TOG protein and are required for granule assembly.

Translation may be granule-dependent because granules contain high local concentrations of translation machinery components [53], which may enhance translation efficiency for RNA molecules in granules compared to RNA molecules freely dispersed in cytosol. In this regard, translation involves several diffusion-dependent steps including: initiation [54], amino-acyl tRNA binding [55] and ribosomal subunit recycling [56] that may be accelerated if components are locally concentrated in granules rather than freely diffusing in cytosol. The results presented here indicate that interfering with granule assembly by disrupting TOG expression does not affect sporadic translation, which is believed to reflect monosomal translation, but reduces bursty translation, which is believed to reflect polysomal translation that may be more sensitive to reduced translational efficiency.

Synaptic function and LTP may be granule-dependent because granule RNAs encode proteins (ARC, αCamKII, NG, PKMξ) that are required for synaptic function and LTP [8], [57]–[62]. Disruption of granule assembly in TOG cKO neurons may reduce translation of these proteins resulting in deficits in synaptic function and LTP.

Hippocampal-associated behaviors such as marble burying, locomotor activity and ultrasonic vocalization may be granule-dependent because they require synaptic function in the hippocampus, which is regulated by proteins encoded by granule RNAs. Decrease in TOG level in the cortex may also contribute to the behavioral deficits by the same mechanism. Stimulus inputs to CA1 hippocampal neurons generate outputs that drive behavior. In control mice continuous stimuli may result in declining output from CA1 hippocampal neurons manifested as short term habituation [32], [46], [63]. Short term habituation may be granule-dependent because output from CA1 hippocampal neurons is regulated by ARC and other proteins encoded by granule-associated RNAs. Thus, in TOG cKO mice impaired granule assembly may result in impaired short term habituation.

TOG cKO mice also emit increased proportions of short high frequency USVs compared to control mice. If this phenotype is also granule-dependent, one or more of the proteins encoded by granule-associated RNAs may encode protein(s) that suppress hippocampal output responsible for emission of high frequency USVs in control mice. Reduced expression of these protein(s) may allow emission of high frequency USVs in TOG cKO mice.

Although most of the phenotypes of TOG cKO mice can be attributed to impaired granule assembly, TOG protein also binds to microtubules, so it is possible that some aspects of the TOG cKO phenotype reflect microtubule-dependent functions. Recent studies indicate that microtubules transiently enter dendritic spines [64], which could affect translation in the post-synaptic compartment [65]. Although microtubule dynamics in TOG cKO neurons was not analyzed directly in this study, the distribution of MAP2, which reflects microtubule organization in dendrites, is similar in TOG cKO and in control neurons, suggesting that microtubule-dependent functions of TOG are not strongly affected. Additional experiments will be necessary to determine if microtubule dynamics in dendritic spines is affected in TOG cKO neurons.

In summary, our results provide the first direct evidence that granule assembly in neurons is required for granule translation, synaptic function, LTP and short term habituation.
